# Regulation of Vascular Smooth Muscle Tone by Adipose-Derived Contracting Factor

**DOI:** 10.1371/journal.pone.0079245

**Published:** 2013-11-11

**Authors:** Matthias R. Meyer, Natalie C. Fredette, Matthias Barton, Eric R. Prossnitz

**Affiliations:** 1 Department of Cell Biology and Physiology, University of New Mexico Health Sciences Center, Albuquerque, New Mexico, United States of America; 2 Molecular Internal Medicine, University of Zürich, Zürich, Switzerland; University of Southampton, United Kingdom

## Abstract

Obesity and arterial hypertension, important risk factors for atherosclerosis and coronary artery disease, are characterized by an increase in vascular tone. While obesity is known to augment vasoconstrictor prostanoid activity in endothelial cells, less is known about factors released from fat tissue surrounding arteries (perivascular adipose). Using lean controls and mice with either monogenic or diet-induced obesity, we set out to determine whether and through which pathways perivascular adipose affects vascular tone. We unexpectedly found that in the aorta of obese mice, perivascular adipose potentiates vascular contractility to serotonin and phenylephrine, indicating activity of a factor generated by perivascular adipose, which we designated “adipose-derived contracting factor” (ADCF). Inhibition of cyclooxygenase (COX) fully prevented ADCF-mediated contractions, whereas COX-1 or COX-2-selective inhibition was only partially effective. By contrast, inhibition of superoxide anions, NO synthase, or endothelin receptors had no effect on ADCF activity. Perivascular adipose as a source of COX-derived ADCF was further confirmed by detecting increased thromboxane A_2_ formation from perivascular adipose-replete aortae from obese mice. Taken together, this study identifies perivascular adipose as a novel regulator of arterial vasoconstriction through the release of COX-derived ADCF. Excessive ADCF activity in perivascular fat under obese conditions likely contributes to increased vascular tone by antagonizing vasodilation. ADCF may thus propagate obesity-dependent hypertension and the associated increased risk in coronary artery disease, potentially representing a novel therapeutic target.

## Introduction

There is growing evidence that perivascular adipose (commonly referred to as perivascular adipose tissue, PVAT), a specific visceral fat compartment that surrounds blood vessels with no fascial layer separating it from the vascular wall, may regulate vascular function through paracrine mechanisms, only some of which have been identified [1]–[4]. Perivascular adipose represents a source of relaxing factors, such as adiponectin, angiotensin 1–7, hydrogen sulfide, and adipose-derived relaxing factor (ADRF) [1]–[4]. On the contrary, little is known about perivascular adipose-derived contractile factors. Stimulated superoxide formation in perivascular adipose, for example, may reduce the bioactivity of the endothelial vasodilator NO [5], thereby indirectly mediating an increase in vascular tone. Whether perivascular adipose releases contracting factors that act directly on vascular smooth muscle has not yet been addressed.

Under healthy conditions, perivascular adipose exerts anti-contractile activity [6]–[9], which is lost in obesity despite concomitant increases in perivascular adipose mass [8]–[10]. This strongly suggests the existence of (yet unidentified) counteracting vasoconstricting mechanisms that become activated when obesity develops, consistent with the notion that increased perivascular adipose mass is associated with arterial hypertension in obese patients [11]. Similarly, we have previously reported that vasoconstriction due to endothelial cell-derived, cyclooxygenase (COX)-dependent prostanoid formation is enhanced in diet-induced and monogenic models of obesity [12]–[14]. However, in these previous studies, perivascular adipose had been removed, excluding the possibility of examining its direct effects on vascular tone.

To address whether perivascular adipose is a source of endogenous vasoconstrictors that might alter the balance between relaxing and contracting factors [15], for the present study we employed not only a diet-induced obesity (DIO) model, but also a novel model of monogenic visceral obesity, the G protein-coupled estrogen receptor (GPER)-deficient mouse [16]–[20]. GPER is a 7-transmembrane G protein-coupled receptor superfamily member that has been shown to mediate many of the rapid physiological and cellular effects of estrogen [16], [17], in conjunction with the classical nuclear estrogen receptors [17]. Animals of both obesity models are normotensive [14], [21], making them particularly attractive to study functional vascular changes associated with the obesity phenotype independent of blood pressure. Similar to changes typically present in animal models of obesity [12], [13], [22] and obese humans [23]–[26], the GPER^0^ obesity model is characterized by visceral obesity [18], [19], [27], dyslipidemia [27], insulin resistance [27] as well as enhanced responses to endothelium-derived vasoconstrictor prostanoids and to endothelin-1 [14], [28]. In view of these findings and given our recent observation that both the GPER^0^ and DIO models exhibit excessive perivascular adipose surrounding the thoracic aorta, we hypothesized that perivascular adipose-derived vasoactive factors might contribute to the regulation of vascular tone in these animals.

The results presented in the present study unexpectedly reveal that perivascular adipose controls arterial smooth muscle tone by releasing an “adipose-derived contracting factor” (ADCF) formed by COX that becomes functionally relevant in obesity. Consistent with a source of COX-derived lipid vasoconstrictors, perivascular adipose releases thromboxane A_2_ in lean mice, and to a greater extent in monogenetic and diet-induced obesity that is likely sufficient to counteract endogenous vasodilator activity.

## Materials and Methods

### Animal models

C57Bl6 (Harlan Laboratories, Indianapolis, IN; 12 months of age) and monogenic obese GPER-deficient (GPER^0^) mice (originally provided by Jan S. Rosenbaum, Proctor & Gamble, Cincinnati, OH, [20]) were bred and housed at the animal research facility of the University of New Mexico Health Sciences Center as described [14], [28]. Only male animals were used to exclude vasoactive and metabolic effects of estrogens [17]. Animals had access to standard rodent chow (16% of total kcal from fat: Teklad Diet 2020SX; Harlan Laboratories) and water *ad libitum*. In a subset of C57Bl6 mice (6 weeks of age), obesity was induced using a 24 week high-fat diet protocol (42% of total kcal from fat: Teklad Diet TD.09821) [29]. All procedures were approved by the University of New Mexico Institutional Animal Care and Use Committee and carried out in accordance with the National Institutes of Health Guide for the Care and Use of Laboratory Animals.

### Blood pressure measurements

Systolic and diastolic blood pressure were measured in conscious mice using a noninvasive volume-pressure monitoring/recording system (CODA-6, Kent Scientific, Torrington, CT), which correlates well with invasive measurements [30]. Following a training period of 5–7 days, means of 16 measurements recorded on 5–8 days were averaged for each mouse and used for subsequent data analysis.

### Isolated vessel preparation and experimental setup

Animals were euthanized by intraperitoneal injection of sodium pentobarbital (2.2 mg/g BW). The thoracic aorta with surrounding perivascular adipose was immediately excised and placed in cold (4°C) physiological saline solution (PSS, composition in mmol/L: 129.8 NaCl, 5.4 KCl, 0.83 MgSO_4_, 0.43 NaH_2_PO_4_, 19 NaHCO_3_, 1.8 CaCl_2_, and 5.5 glucose; pH 7.4). Isolated thoracic aortae were cut into 4 mm long segments, which were divided into two 2 mm long rings, where perivascular adipose was either carefully removed or left intact. This allowed for direct study of the effects of perivascular adipose within the same segment of one thoracic aorta. Vessels were transferred to an organ chamber of a Mulvany-Halpern myograph [31] and mounted onto two 200 µm pins, one connected to a force transducer and the other to a micropositioner (620M multi-channel myograph, Danish Myo Technology, Aarhus, Denmark) as described [14].

### Vascular function analysis

After equilibrating in PSS (37°C; oxygenated with 21% O_2_, 5% CO_2_, and balanced N_2_; pH 7.4 [32]) for 30 min, rings were progressively stretched to the optimal passive tension for generating force during isometric contraction (19 mN) [14]. Rings were allowed to equilibrate for 45 min, and repeatedly exposed to KCl (PSS with equimolar substitution of 60 mmol/L potassium for sodium) until a stable response was achieved. Rings that developed less than 9.81 mN of force were discarded. Where indicated, rings were incubated with either the cyclooxygenase (COX) inhibitor meclofenamate (1 µmol/L), the COX-1-selective inhibitor SC-560 (300 nmol/L), the COX-2-selective inhibitor CAY10404 (100 nmol/L), the NO synthase inhibitor L-N^G^-nitroarginine methyl ester (L-NAME, 300 µmol/L), the superoxide dismutase mimetic 1-oxyl-2,2,6,6-tetramethyl-4-hydroxypiperidine (Tempol, 100 µmol/L), or the highly selective ET_A_ endothelin receptor antagonist BQ-123 (100 nmol/L) [33] for 30 min prior to exposure to agonists. Subsequently, concentration-response curves to serotonin (1 nmol/L – 10 µmol/L) or phenylephrine (1 nmol/L – 1 µmol/L) were obtained.

### Quantitation of thromboxane A_2_ release by perivascular adipose

The *ex vivo* formation of thromboxane A_2_ from perivascular adipose was assayed using a competitive enzyme immunoassay (EIA) as described [34]. Briefly, after incubating neighboring aortic rings with and without perivascular adipose with the highest serotonin concentration (10 µmol/L) for 3 min, the bath fluid (5 mL) was snap-frozen in liquid nitrogen and stored at −80°C for subsequent analysis. Thromboxane A_2_ production was measured by determining the concentration of its hydrolyzed metabolite, thromboxane B_2_ [35], using a competitive EIA kit according to the manufacturer's instructions (Cayman Chemical, Ann Arbor, MI, USA). In order to quantify thromboxane production specifically originating from perivascular adipose, individual aortic segments (4 mm in length) were divided into two equally sized rings, and thromboxane production was determined in one half with perivascular adipose removed. This value was subtracted from that measured in the other half with intact perivascular adipose, and values were normalized to tissue dry weight.

### Quantitation of perivascular adipose steady-state mRNA expression levels

Aortic perivascular adipose (30mg) was snap-frozen in liquid nitrogen and disrupted using a rotor-stator homogenizer. Total RNA was extracted using the silica-based RNeasy Lipid Tissue Mini Kit (Qiagen, Valencia, CA, USA). RNA was reverse transcribed with the High-Capacity cDNA Reverse Transcription Kit (Applied Biosystems, Carlsbad, CA, USA). PCR was performed using SYBR Green-based detection of amplified gene-specific cDNA fragments on a 7500 FAST real-time PCR System (Applied Biosystems) using the sets of primers given in Table S1, with specificity confirmed by melting curve analysis. Gene expression was calculated based on the 2^−ΔΔCT^ method [36]. GAPDH served as a housekeeping control and showed identical C_T_ values between groups.

### Substances and drugs

Meclofenamate, SC-560, CAY10404 and L-NAME were from Cayman Chemical. Serotonin was from MP Biomedicals (Solon, OH, USA), and Tempol from Tocris Bioscience (Minneapolis, MN, USA). All other drugs were from Sigma-Aldrich (St. Louis, MO, USA). Stock solutions were prepared according to the manufacturer's instructions, and diluted in PSS to the required concentrations before use. Concentrations are expressed as final molar concentration in the myograph chamber.

### Data calculation and statistical analyses

Contraction is given as the percentage of contraction relative to 60 mM KCl [14]. Maximal responses, area under the curve (AUC) and EC_50_ values (expressed as pD_2_) of the concentration-response curves were calculated by curve fitting as described by deLean *et al.* [37], using FitLab® software run in MatLab 5.0 on a Macintosh computer. Where no plateau was reached, AUC was calculated using the trapezoidal rule approximation in Prism (version 5.0 for Macintosh, GraphPad Software, San Diego, CA, USA) [38]. Parametric data were analyzed using the two-tailed Student's *t* test or by two-way ANOVA followed by Bonferroni's post-hoc test as appropriate; non-parametric data were analyzed using the Mann-Whitney *U* test (Prism version 5.0 for Macintosh). All data are expressed as mean ± SEM. A *p* value of <0.05 was considered significant.

## Results

### Monogenic obesity increases perivascular adipose mass

**Figure 1:**
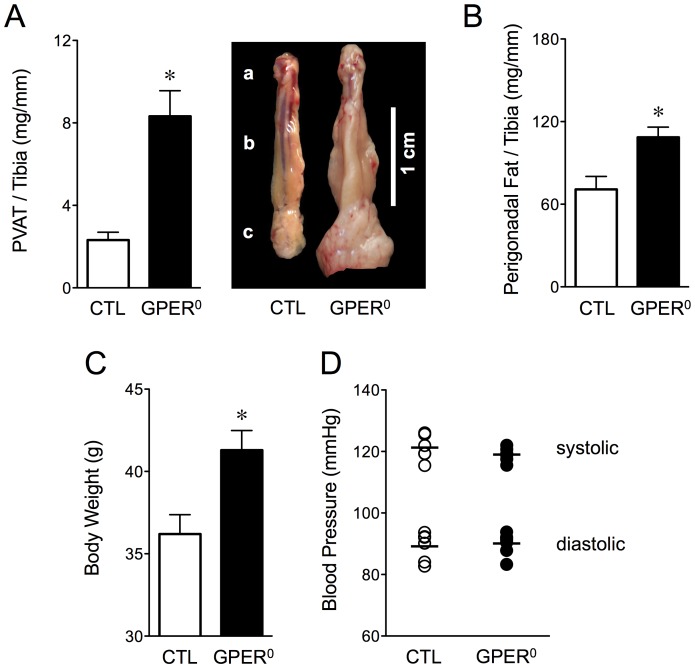
Perivascular adipose and perigonadal fat mass, body weight and blood pressure in monogenic obesity (GPER^0^). A, Mass and macroscopic difference in the quantity of perivascular adipose (PVAT) surrounding the aorta (a, Aortic arch; b, Thoracic aorta; c, Abdominal aorta) of obese (GPER^0^, *n* = 5) and lean WT control mice (CTL, *n* = 8). B, Perigonadal fat weight (CTL, *n* = 8; GPER^0^, *n* = 9); C, body weight (*n* = 19/group); D, systolic and diastolic blood pressure levels in obese (GPER^0^, 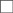, *n* = 7) and lean WT mice (CTL, 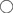, *n* = 6). Fat weights are normalized to tibial length. A–C: open bars, lean WT control (CTL); solid bars, monogenic obesity (GPER^0^). *p<0.01 *vs.* control.

In preliminary experiments with the GPER^0^ model of monogenic obesity [18], [19], we observed a marked increase in perivascular adipose mass compared with WT controls (3.6-fold increase, adipose mass normalized to tibial length, 8.3±1.2 mg/mm *vs.* 2.3±0.4 mg/mm, *n* = 5–8, *p*<0.01 *vs.* control, Figure 1A). By contrast, the increase in perigonadal fat mass in GPER^0^ mice was less pronounced (1.6-fold, normalized to tibial length, 108.6±7.4 *vs.* 70.8±9.4 mg/mm, *n* = 8–9, *p*<0.01 *vs.* control, Figure 1B). The increase in visceral fat was associated with a significant increase in body weight (41.3±1.2 *vs.* 36.2±1.2 g, *n* = 19, *p*<0.01 *vs.* lean WT control, Figure 1C), whereas tibial length as a measure of body length was identical in GPER^0^ and WT control animals (18.6±0.1 mm, *n* = 5). Obesity (body weight and perivascular adipose) was already present in GPER^0^ animals at 3 months of age (Figure S1), but by 12 months of age had progressed much further in adult animals (Figure 1). Therefore, 12 month-old animals were selected for vascular function analyses. Despite excessive visceral fat accumulation [18], [27], blood pressure in GPER^0^ mice remained unchanged compared to controls (systolic, 119.0±0.8 *vs.* 121.3±1.7 mmHg; diastolic, 90.2±1.3 *vs.* 89.3±1.9 mmHg; *n* = 6–7, Figure 1D), consistent with normotensive blood pressure levels measured in the DIO model [21].

Murine perivascular adipose from the aorta is highly similar to brown adipose tissue and resistant to obesity-induced inflammation [39]. Accordingly, steady-state gene expression levels of the pro-inflammatory tumor necrosis factor (TNF)-α and leptin genes in perivascular adipose were unaffected by GPER deficiency (Table 1). We also found no change in peroxisome proliferator activated receptor PPARγ expression (Table 1). Of note, expression levels of adiponectin (which has been designated a perivascular adipose-derived relaxing factor [ADRF] in humans [9], but not in mice [8]), was reduced 2.4-fold in perivascular adipose from GPER^0^ mice (Table 1).

**Table 1:** Quantitative measurements of adipocyte-related gene expression in aortic perivascular adipose from GPER^0^ and control mice.

	Control	GPER^0^	*p* Value
Adiponectin	1.7±0.3	0.7±0.2	0.04
Leptin	88.2±36.7	89.7±18.6	0.97
TNF-α	5.4±2.3	5.6±3.7	0.95
PPARγ	292.0±65.2	213.1±29.2	0.30

Adipose was collected and analyzed from animals with monogenic (GPER^0^) obesity and compared to lean WT controls (*n* = 5/group). Expression levels of mRNA were calculated based on the 2^−ΔΔCT^ method and expressed as arbitrary units. GAPDH served as the housekeeping control.

### Perivascular adipose potentiates serotonin-induced contractions in monogenic obesity

**Figure 2:**
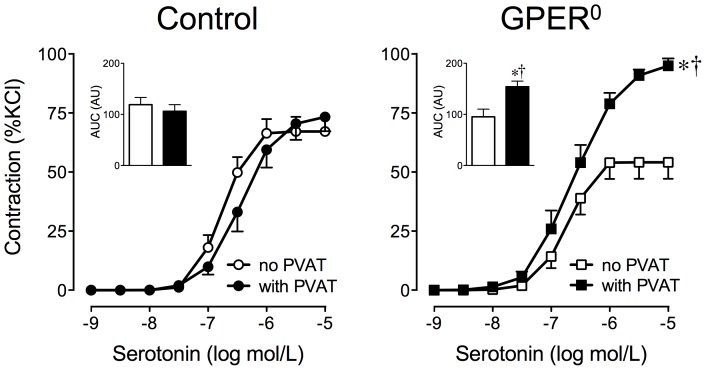
Potentiating effect of perivascular adipose on serotonin-induced contractions in mice with monogenic obesity. Aortic rings with and without perivascular adipose (PVAT) from monogenetic obese GPER^0^ or lean WT (control) mice were exposed to increasing concentrations of serotonin. Inset: Area under the curve (AUC) of the concentration-response curves is expressed as arbitrary units (AU). 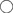, control without PVAT (*n* = 7); 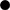, control with PVAT (*n* = 6); 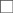, GPER^0^ without PVAT (*n* = 6); 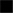, GPER^0^ with PVAT (*n* = 7). *p<0.05 vs. aortic rings without perivascular adipose; †*p*<0.05 *vs.* control.

We hypothesized that perivascular adipose might alter agonist-stimulated contractility, which is enhanced in obesity [12]–[14]. In perivascular adipose-intact aortic rings of mice with monogenic obesity (GPER^0^), maximal contractions to serotonin (relative to 60 mmol/L KCl) were enhanced (1.3-fold, 93.8±2.8 *vs.* 73.2±6.1%KCl, *n* = 6–7, *p*<0.01 *vs.* control, Figure 2). Removal of perivascular adipose markedly reduced maximal serotonin-induced contractions in rings from GPER^0^ obese mice (47% inhibition, from 93.8±2.8 to 54.4±7.0%KCl, *n* = 6–7, *p*<0.001 *vs.* rings without adipose) but not in lean WT controls (73.2±6.1 *vs.* 67.4±6.0%KCl, *n* = 6–7, *p* = n.s. *vs.* rings without adipose, Figure 2). Furthermore, contractions to serotonin in rings without perivascular adipose were not different between mice with monogenic obesity and WT controls (Figure 2). Moreover, KCl induced similar responses in rings with and without perivascular adipose of both GPER^0^ mice (16.1±0.7 *vs.* 16.3±0.8 mN) and controls (16.4±0.5 *vs.* 16.0±0.5 mN).

In lean WT controls, the sensitivity of serotonin-induced contractions was slightly but significantly lower when perivascular adipose was present (pD_2_ 6.40±0.12 *vs.* 6.72±0.06 umol/L, *n* = 6–7, *p*<0.05 *vs.* rings without perivascular adipose), indicative of an *anti-contractile* effect of perivascular adipose in lean animals (Figure 2). These findings are consistent with previous observations in the murine aorta [40], whereas perivascular adipose-mediated anti-contractile effects are substantially greater in rats [7], [8]. However, our finding of potentiation of contractions by perivascular adipose in mice with monogenic obesity were the first suggestion that a *contracting* factor derived from perivascular adipose increases vascular tone by potentiating serotonin-induced contractions. Due to its site of origin this factor was termed “adipose-derived contracting factor” (ADCF).

### Functional characterization of adipose-derived contracting factor (ADCF)

**Figure 3:**
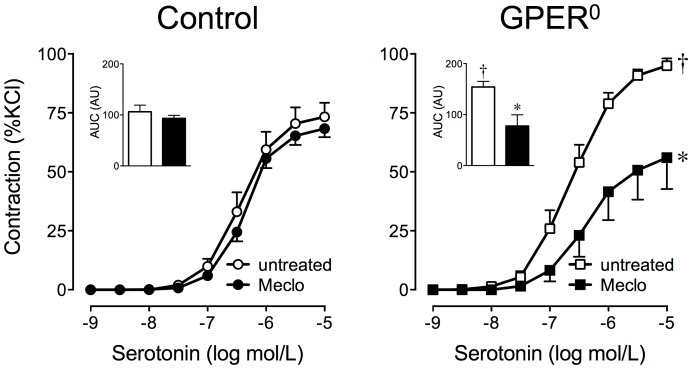
Cyclooxygenase inhibition prevents perivascular adipose-dependent potentiation of contractions to serotonin in monogenic obesity. Aortic rings with perivascular adipose (PVAT) from monogenic obese GPER^0^ mice or lean WT (control) mice were pretreated with the cyclooxygenase inhibitor meclofenamate (Meclo, 1 µmol/L) prior to stimulation with increasing concentrations of serotonin. Inset: Area under the curve (AUC) of concentration-response curves is expressed as arbitrary units (AU). 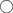, control, untreated (*n* = 6); 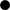, control, meclofenamate (*n* = 7); 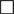, GPER^0^, untreated (*n* = 7); 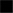 GPER^0^, meclofenamate (*n* = 6). *P<0.001 *vs.* untreated vascular rings; †*p*<0.05 *vs.* control.

We have previously reported that obesity activates the formation of cyclooxygenase (COX)-derived vasoconstrictor prostanoids in endothelial cells [12]–[14]. Given that adipocytes as well as other cell types present in adipose express COX [41], [42], we hypothesized that the ADCF-dependent potentiation of serotonin-induced contractions might be mediated by COX products. Gene expression analyses detected both COX-1 and COX-2 isoforms in perivascular adipose, with steady-state mRNA expression levels of COX-1 being about 80-fold higher than those of COX-2 (Table 2). Monogenic obesity had no effect on mRNA expression levels (Table 2). Since a lack of change in gene or even protein expression does not rule out changes in enzyme activity, we determined whether COX-derived vasoconstrictor prostanoids contribute to the potentiation of serotonin-induced contractions by treating perivascular adipose-intact rings with the isoform-nonselective COX-inhibitor meclofenamate (1 µmol/L for 30 min). In aortic rings of mice with monogenic obesity, inhibition of COX completely prevented perivascular adipose-dependent contractile effects, reducing serotonin-induced contractions by 41% (from 93.8±2.8 to 54.9±13%KCl, *n* = 5–7, *p*<0.01 *vs.* untreated rings, Figure 3). By contrast, in lean WT controls, COX inhibition had no effect on contractions to serotonin (67.4±3.5 *vs.* 73.2±6.1%KCl, *n* = 6–7, *p* = n.s. *vs.* untreated rings, Figure 3).

**Table 2:** Effect of obesity on gene expression of cyclooxygenase (COX)-1 and COX-2 isoforms in aortic perivascular adipose.

	Monogenic Obesity		Diet-induced Obesity	
	Control	GPER^0^	Control	DIO
COX-1	70±13	61±14	87±27	89±16
COX-2	0.8±0.2*	0.6±0.1*	1.1±0.3*	1.2±0.3*

Adipose was collected and analyzed from animals with monogenic (GPER^0^, *n* = 5) obesity or in diet-induced obesity (DIO, *n* = 6), as well as from lean, age-matched WT controls (*n* = 6). Expression levels of mRNA were calculated based on the 2^−ΔΔCT^ method and expressed as arbitrary units. GAPDH served as housekeeping control. *p<0.01 *vs.* COX-1.

To exclude an effect specific to serotonin as an agonist, parallel experiments were performed with the predominantly α_1_-adrenergic agonist phenylephrine. As with serotonin-induced contractions, phenylephrine-induced contractions were equally reduced by COX inhibition in aortic rings with intact perivascular adipose from obese mice (41% inhibition of the 1 µM phenylephrine response, from 77.3±2.1 to 46.0±3.8%KCl, *n* = 6, *p*<0.001 *vs.* untreated rings), but not in lean controls (Figure S2), indicating that ADCF becomes activated in obesity and augments contractions regardless of the contractile agonist.

To further delineate which COX isoform mediates the ADCF effect in serotonin-induced contractions, we performed experiments using either the COX-1-selective inhibitor SC-560 or the COX-2-selective inhibitor CAY10404. While meclofenamate fully prevented ADCF activity (Figure 3, right panel), each isoform-selective COX inhibitor was only partially effective (Figure S3). By contrast, SC-560 and CAY10404 had no effect on rings from healthy, lean mice (Figure S3).

In order to rule out the possible contribution of additional factors in the adipose-mediated potentiation of contraction, experiments were performed to assess other vasoconstrictor pathways and targets known to play a role in vascular disease development and obesity, such as endothelin-1 [43] and reactive oxygen species [22], [25], [44]. None of the following inhibitors had an effect on ADCF activity during serotonin-induced contractions in rings with perivascular adipose from obese mice: the NO synthase inhibitor L-NAME, as uncoupled NO synthase can produce vasoconstricting superoxide [45] (93.1±2.9 *vs.* 93.8±2.8%KCl, *p* = n.s.), the superoxide scavenger and superoxide dismutase mimetic Tempol [46] (89.5±2.9 *vs.* 93.8±2.8%KCl, *p* = n.s.) or the highly ET_A_-selective endothelin receptor antagonist BQ-123 [33] (88.4±2.6 *vs.* 93.8±2.8%KCl, *p* = n.s.). These results exclude a contribution of these pathways in the ADCF responses observed and thus further corroborate the nature of ADCF as a COX-derived vasoconstrictor prostanoid in monogenetic obesity.

### Effect of perivascular adipose on vascular tone in diet-induced obesity

**Figure 4:**
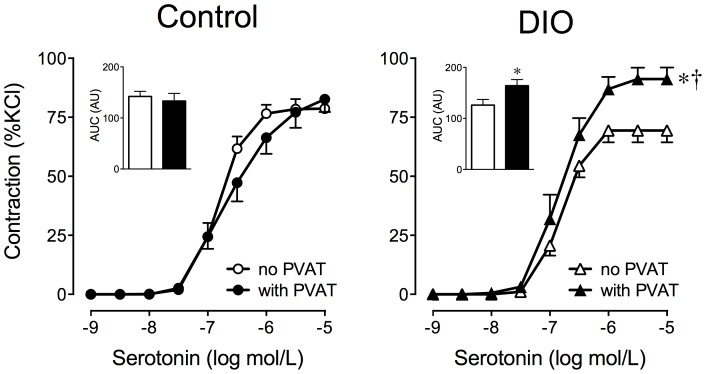
Effect of perivascular adipose on serotonin-induced contractions in mice with diet-induced obesity. Aortic rings with and without perivascular adipose (PVAT) from WT mice with diet-induced obesity (DIO) or lean WT mice (control) mice were exposed to increasing concentrations of serotonin. DIO animals were fed a high-fat diet for 24 weeks and compared to age-matched mice fed a standard chow (control). Inset: Area under the curve (AUC) is expressed as arbitrary units (AU). 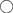, control, without PVAT (*n* = 6); 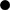, control, with PVAT (*n* = 6); 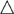, DIO, without PVAT (*n* = 5); 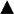, DIO, with PVAT (*n* = 7). *p<0.05 *vs.* aortic rings without perivascular adipose; †*p*<0.05 *vs.* control.

In order to rule out extraneous effects of GPER deficiency by solely using the monogenic GPER^0^ model of obesity, we also characterized the effect of perivascular adipose on contractions in the well-established C57BL6 model of diet-induced obesity (DIO) [29]. Similar to the monogenic GPER^0^ obesity model, DIO mice showed an increase in perivascular adipose mass (2-fold, adipose mass normalized to tibial length, 7.0±0.7 *vs.* 3.5±0.3 mg/mm, *n* =  6–10, *p*<0.01 *vs.* control), perigonadal fat mass (1.6-fold, normalized to tibial length, 141.3±5.4 *vs.* 87.4±10.6 mg/mm, *n* = 6–10, *p*<0.001 *vs.* control) and body weight (42.9±1.0 *vs.* 35.9±1.6 g, *n* = 9–12, *p*<0.01 *vs.* control). Consistent with the ADCF-mediated potentiation of serotonin-induced contractions in monogenic obesity (Figure 2), contractions in perivascular adipose-intact rings were increased by 29% in DIO mice (90.1±4.5 *vs.* 69.9±5.1%KCl, *n* = 5–7, *p*<0.01 *vs.* rings without perivascular adipose, Figure 4). The presence of perivascular adipose had no effect on contractions in age-matched lean controls (80.2±5.1 *vs.* 78.3±3.8%KCl, *n* = 6, *p* = n.s. *vs.* rings without perivascular adipose, Figure 4). Of note, KCl-induced contractions in rings with and without perivascular adipose were similar in DIO (14.3±0.5 *vs.* 13.8±1.1 mN) and control mice (14.2±0.3 *vs.* 13.9±0.3 mN).

**Figure 5:**
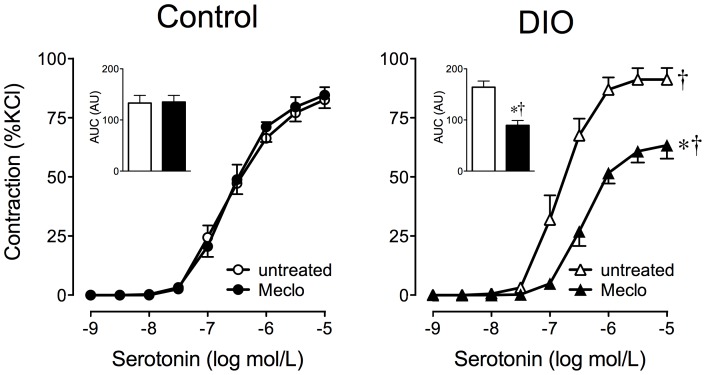
Cyclooxygenase inhibition prevents perivascular adipose-dependent potentiation of contractions to serotonin in diet-induced obesity. Aortic rings with perivascular adipose (PVAT) from WT mice with diet-induced obesity (DIO) or lean WT mice (control) mice were exposed to increasing concentrations of serotonin in the presence or absence of the cyclooxygenase inhibitor meclofenamate (Meclo, 1 µmol/L). DIO animals were fed a high-fat diet for 24 weeks and compared to age-matched mice fed a standard chow (control). Inset: Area under the curve (AUC) is expressed as arbitrary units (AU). 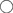, control, untreated (*n* = 6); 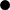 control, meclofenamate (*n* = 4); 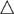, DIO, untreated (*n* = 7); 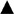, DIO, meclofenamate (*n* = 5). *p<0.001 *vs.* untreated vascular rings; †*p*<0.05 *vs.* control.

We next determined the effect of the COX inhibitor meclofenamate on the ADCF response with serotonin-mediated contractions in DIO mice. Incubation with meclofenamate markedly reduced contractions to levels that were even below those of untreated controls (62.2±5.1 *vs.* 90.1±4.5%KCl, *n* = 5–7, *p*<0.01 *vs.* untreated rings, Figure 5). Meclofenamate had no effect on rings from lean mice (82.6±5.3 *vs.* 80.2±5.1%KCl, *n* = 4–6, *p* = n.s. *vs.* untreated rings, Figure 5). Similar to the results obtained with the monogenic obesity model (Figure S3), the COX-1-selective and COX-2-selective inhibitors each only partially attenuated the ADCF effect in DIO mice, but had no effect in lean controls (Figure S4). As in monogenic obesity, the ADCF effect on contractions to phenylephrine in DIO mice was also reduced considerably by nonselective COX inhibition (20% inhibition of the 1 µM phenylephrine response, from 91.4±2.4 to 74.1±3.8%KCl, *n* = 6, *p*<0.01 vs. untreated rings, Figure S2). However, there was no difference in perivascular mRNA expression levels of COX-1 or COX-2 between DIO mice and lean controls (Table 2). The results of these experiments provide further evidence that a COX-derived ADCF becomes functionally active in obesity, regardless of the cause (i.e. primary obesity due to gene mutation or secondary obesity due to excessive caloric intake). Thus, ADCF activation appears to represent a general functional change that may be due to conditions, such as obesity, that are, at least in part, associated with an increase in overall and/or perivascular adipose mass.

### Chemical nature of the ADCF activity released from perivascular adipose

**Figure 6:**
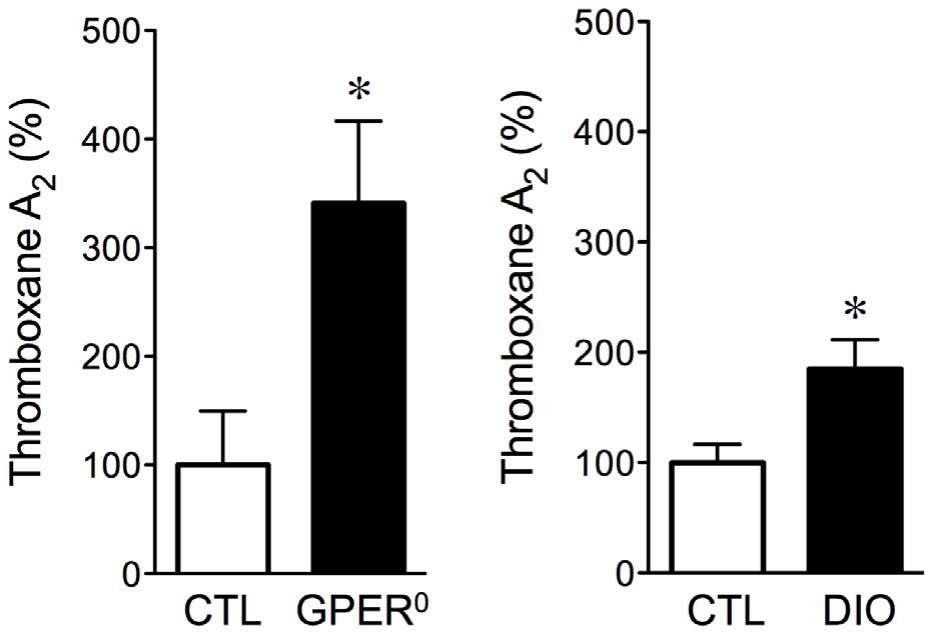
Production of thromboxane A_2_ by perivascular adipose. Thromboxane A_2_ release was stimulated during serotonin-dependent contractions and measured as its hydrolyzed metabolite thromboxane B_2_. Measurements in vascular rings without perivascular adipose were subtracted from rings with perivascular adipose and normalized to dry weight to reveal functional changes in perivascular adipose. Measurements were performed in perivascular adipose from mice with monogenic obesity (GPER^0^, left panel, solid bar, *n* = 4) or from mice with diet-induced obesity (DIO, right panel, solid bar, *n* = 5). Age-matched WT mice fed a regular chow served as controls (CTL, open bars, *n* = 6, mean thromboxane A_2_ production: 15.1±3.8 pg/mg). *p<0.05 *vs.* CTL.

The results of the functional experiments obtained in mice with monogenic obesity and DIO strongly suggested that the ADCF activity released from perivascular adipose might consist of lipid-based vasoconstrictor prostanoids. Using the bioassay as a source of adipose-derived factors, we determined the generation of COX-dependent products in perivascular adipose by measuring the amount of the predominant COX-derived vasoconstrictor prostanoid thromboxane A_2_ [15], [42] released in response to serotonin-mediated contractions. Surprisingly, thromboxane A_2_ was produced by the perivascular adipose of lean control mice (Figure 6). However, both monogenic obesity and DIO were associated with greatly increased thromboxane A_2_ production in adipose (241% and 85% increases, respectively, normalized to perivascular adipose weight, *n* = 4–6, *p*<0.05 *vs.* control, Figure 6). These findings are compatible with the concept that perivascular adipose-released ADCF activity consists of COX-derived vasoconstrictor prostanoids, including thromboxane A_2_. ADCF may act via paracrine effects on vascular smooth muscle to increase vascular tone through COX-derived contracting factors such as thromboxane A_2_, prostaglandin D_2_, prostaglandin E_2_, prostaglandin F_2_α, prostaglandin H_2_, or prostaglandin I_2_ (prostacyclin) [15], [47].

## Discussion

A role for perivascular adipose as a source of neurohumoral regulators was first proposed more than 20 years ago, when Soltis and Cassis reported an indirect vasodilator activity [6]. Subsequent studies have shown that perivascular adipose releases several adipokines capable of reducing vascular tone under healthy conditions [1]–[4], [7]–[9]. Using models of monogenic obesity [14] and DIO [12], [13], we have previously reported increased cyclooxygenase (COX)-dependent endothelial prostanoid formation in arterial rings devoid of perivascular adipose. Abnormal COX-mediated vasoconstriction has also been confirmed in obese humans [25], [26]. The role of perivascular adipose in the regulation of vasoconstrictor tone, both under basal conditions as well as in obesity, is less clear [48]. Previous observations that the anti-contractile activity of perivascular adipose [6]–[9] is lost in obesity despite an increase in net perivascular adipose mass [8]–[10] strongly suggested the existence of (yet unidentified) counteracting vasoconstrictor mechanisms that become activated only as obesity develops. The present study now identifies perivascular adipose as a mediator of increased arterial smooth muscle tone through the release of COX-derived vasoconstrictor prostanoids, which we have designated “adipose-derived contracting factor” (ADCF). We also found that perivascular adipose releases the COX-derived vasoconstrictor thromboxane A_2_ in response to serotonin in healthy, lean mice. Of note, thromboxane A_2_ formation from perivascular adipose is greatly enhanced both in primary (genetic) GPER^0^ obesity [18], [19], and secondary obesity following excess caloric intake [29]. The results indicate that perivascular adipose is a novel source of COX-derived vasoconstrictor activity that increases vascular smooth muscle tone, demonstrating enhanced effects in obesity.

Since the 1930s, tissue has been used to characterize and identify vasoactive factors controlling smooth muscle tone [49], [50]. Similar to the COX-dependent ADCF activity identified from perivascular adipose reported in the present study, a smooth muscle vasoconstrictor activity released from rat lungs [49], [50] was later shown to be sensitive to COX inhibition [51]–[53]. Because the “active non-histamine material” [50] also contracted arterial blood vessels, Vane and coworkers renamed this activity “*rabbit aortic contracting substance*” (RCS) [53], [54]. RCS was subsequently shown to be derived from a microsomal enzyme [55] identical to COX and to evoke contraction predominantly through the prostaglandin H_2_ derivative, thromboxane A_2_ [56], which was also found to potently activate platelets [35]. Shortly thereafter, prostaglandin H_2_/thromboxane A_2_ were identified as COX-derived endothelial cell prostanoids mediating smooth muscle contraction via paracrine mechanisms (endothelium-derived contracting factors, EDCF) [15], [47], [57]. In the present study, we now demonstrate for the first time that COX-derived constrictor activity mediating vascular smooth muscle contraction is also produced by perivascular adipose.

It has previously been shown that, when a patient becomes obese, perivascular adipose loses its anti-contractile activity normally present in small gluteal arteries [9]. Similar results have been reported in mesenteric arteries with the onset of monogenic obesity in mice [8], whereas no such effect is observed in the aorta of diet-induced obese rats [10]. These studies, none of which further characterized the identity of the contractile factor(s) derived from perivascular adipose, suggest a profound heterogeneity in perivascular adipose-dependent regulation of arterial vasoconstriction that may depend on the vascular bed and species studied. Furthermore, perivascular adipose surrounding the murine aorta is highly similar to brown fat and resistant to inflammatory activation in response to a high-fat diet, and therefore differs from other vascular beds [39]. Anatomic heterogeneity might explain the ADCF activity present in murine aorta reported in the present study, which, unlike murine mesenteric resistance arteries or rat aorta [6]–[8], [10], displays only small adipose-dependent anti-contractile effects under healthy conditions [40].

Obesity results in increased activity of COX-1 and its inducible isoform COX-2 in adipose [42], key enzymes involved in the generation of prostaglandins, prostacyclins and thromboxanes, including the potent vasoconstrictor thromboxane A_2_ [42]. Different cell types present in perivascular adipose such as adipocytes, endothelial cells, fibroblasts, and immune cells, express COX and thus may contribute to ADCF formation [42]; in particular, adipocytes have been found to express both COX-1 and COX-2 [41]. Cell-specific deletion of COX isoforms might allow further characterization of the cellular source(s) of ADCF activity in perivascular adipose. Of note, both isoform-selective inhibitors for COX-1 and COX-2 were each partially effective in counteracting ADCF activity. The ADCF response contrasts the enhanced EDCF activity in obesity (in arteries devoid of perivascular adipose [12]–[14], [58], [59]), which is strictly COX-1 dependent [12], [58], [60]. Taken together, obesity appears to involve enhanced activity of both *endothelium*-derived and *perivascular adipose*-derived vasoconstrictor prostanoids further lending support to the concept that COX is centrally involved in the enhanced net vasoconstriction observed in obesity in both animals and humans [22], [26].

It is currently not known what mechanisms trigger formation of ADCF in obesity; however, this may be functionally related to or involve local hypoxia. Rapid adipose expansion in obesity, including perivascular adipose, may cause local hypoxia [61], resulting in dysregulated production of adipokines within perivascular adipose [62]. Furthermore, in obesity, hypoxia may also increase non-adipose, i.e. vascular production of vasoconstrictor prostanoids [59]. In agreement with our findings obtained in two different models of obesity, hypoxia similarly reduces anti-contractile properties of perivascular adipose [9]; acute hypoxia even results in transient contractions that are partly COX-dependent [2]. These findings support the concept that in obesity both perivascular adipose and endothelial cells represent sources of COX-derived vasoconstrictor prostanoids, including thromboxane A_2_, which may act in concert to increase vascular smooth muscle tone. Finally, the discovery of ADCF activity identifies perivascular adipose as a novel regulator of vasoconstriction that may help to explain blood pressure changes in genetically modified mice. For example, deletion of the thromboxane prostanoid (TP) receptor, the main target of vasoconstrictor prostanoids [47], is associated with lower systolic blood pressure than in wild-type mice [63]. This may be due, at least in part, to a lack of contraction via perivascular adipose-derived prostanoids, including thromboxane A_2_. Moreover, a vasoconstrictor role for perivascular adipose is further supported by data obtained in mice with smooth muscle-specific deletion of PPARγ [64], [65]. These mice are characterized by a complete lack of perivascular adipose and are hypotensive during their resting period [64], [65].

In summary, the present study has identified and characterized an “adipose-derived contracting factor” (ADCF) formed through COX-dependent pathways in perivascular adipose and detected formation of the potent vasoconstrictor thromboxane A_2_ from perivascular adipose in lean and, to a greater extent, obese mice. The functional activity of this ADCF was shown to contribute to the increased vascular tone in experimental obesity and may also play a role in the increased vascular tone in obese patients [11]. Whether therapeutic countermeasures, such as physical exercise or reducing dietary calorie intake, both of which improve endothelial-dependent vasodilator function [22], [66], might inhibit ADCF activity or increase perivascular adipose-dependent vasodilator capacity, remains to be determined. The recent finding that in obese patients, augmented vasoconstriction can be blocked by a COX-inhibitor [26] suggests that release and activity of ADCF could, at least in part, contribute to the increased vascular smooth muscle tone and thus development of hypertension in obese humans. Pharmacological inhibition of COX-dependent ADCF formation or reducing perivascular adipose mass, secondary to weight reduction, may thus represent novel therapeutic strategies to lower vascular tone and cardiovascular disease risk associated with obesity. Finally, Bailey et al. [67] and FitzGerald and colleagues [68] have demonstrated that COX-derived prostanoids directly contribute to atherosclerosis progression. Thus, the recently reported observation that perivascular adipose aggravates atherosclerosis [69] suggests the involvement of ADCF-dependent mechanisms.

## Supporting Information

Figure S1
**Obesity in 3 month-old male GPER^0^ mice.** A, Body weight of 3 month-old male GPER^0^ mice (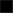, *n* = 16) and WT controls (CTL, 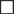, *n* = 27). *p = 0.01 vs. CTL. B, Aortic perivascular adipose (PVAT) mass of 3 month-old male GPER^0^ (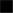, *n* = 5) and CTL mice (CTL, 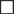, *n* = 5). *p = 0.02 *vs.* CTL.(TIFF)

Figure S2
**COX inhibition of phenylephrine-induced contractions in vessels of obese mice.** Aortic rings with perivascular adipose were obtained from WT mice fed a regular chow (control), GPER^0^ mice, and WT animals fed a high-fat diet (DIO). Where indicated, rings were treated with the cyclooxygenase inhibitor meclofenamate (Meclo, 1 mmol/L) prior to stimulation. Inset: Area under the curve (AUC) is expressed as arbitrary units (AU). 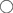, control, untreated (*n* = 6); 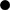, control, meclofenamate (*n* = 7); 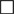, GPER^0^, untreated (*n* = 6); 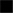 GPER^0^, meclofenamate, (*n* = 6); 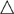, DIO, untreated (*n* = 6); 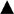, DIO, meclofenamate (*n* = 6). *p<0.01 *vs.* untreated vascular rings; †*P*<0.05 *vs.* control.(TIFF)

Figure S3
**Cyclooxygenase subtype-specific inhibition of vascular contraction in mice with monogenic obesity.** Aortic rings with perivascular adipose were obtained from GPER^0^ mice and WT mice fed a regular chow (control). Concentration-dependent contractions to serotonin were determined in the presence of selective inhibitors for cyclooxygenase type 1 (SC-560, 300 nmol/L) and type 2 (CAY10404, 100 nmol/L). Inset: Area under the curve (AUC) is expressed as arbitrary units (AU). 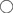, control, untreated (*n* = 6); 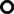, control, SC-560 (*n* = 4); 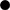, control, CAY10404 (*n* = 4); 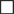, GPER^0^, untreated (*n* = 7); 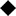, GPER^0^, SC-560 (*n* = 7); 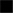 GPER^0^, CAY10404 (*n* = 6). *p<0.05 *vs.* untreated vascular rings; †*p*<0.05 *vs.* control.(TIFF)

Figure S4
**Cyclooxygenase subtype-specific inhibition of vascular contraction in perivascular adipose-intact aortic rings of mice with diet-induced obesity.** Animals were fed a high-fat diet for 24 weeks (DIO) and compared to age-matched mice fed standard chow (control). Concentration-dependent contractions to serotonin were determined in the presence of selective inhibitors for cyclooxygenase type 1 (SC-560, 300 nmol/L) and type 2 (CAY10404, 100 nmol/L). Inset: Area under the curve (AUC) is expressed as arbitrary units (AU). 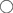, control, untreated (*n* = 6); 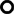, control, SC-560 (*n* = 4); 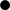, control, CAY10404 (*n* = 4); 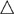, DIO, untreated (*n* = 7); 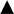, DIO, SC-560 (*n* = 6); 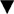, DIO, CAY10404 (*n* = 5). *p<0.05 *vs.* untreated vascular rings; †*p*<0.05 *vs.* control.(TIFF)

Table S1 **Sets of primers used for amplification of gene-specific cDNA fragments by qPCR.** TNFα, tumor necrosis factor α; PPARγ, peroxisome proliferator activated receptor γ; COX, cyclooxygenase.
(DOC)
