# Supplementary figures and images for: Regulation of Vascular Smooth Muscle Tone by Adipose-Derived Contracting Factor
Source: PLoS One. 2013 Nov 11;8(11):e79245. doi: 10.1371/journal.pone.0079245 (PMC3823600; doi:10.1371/journal.pone.0079245)

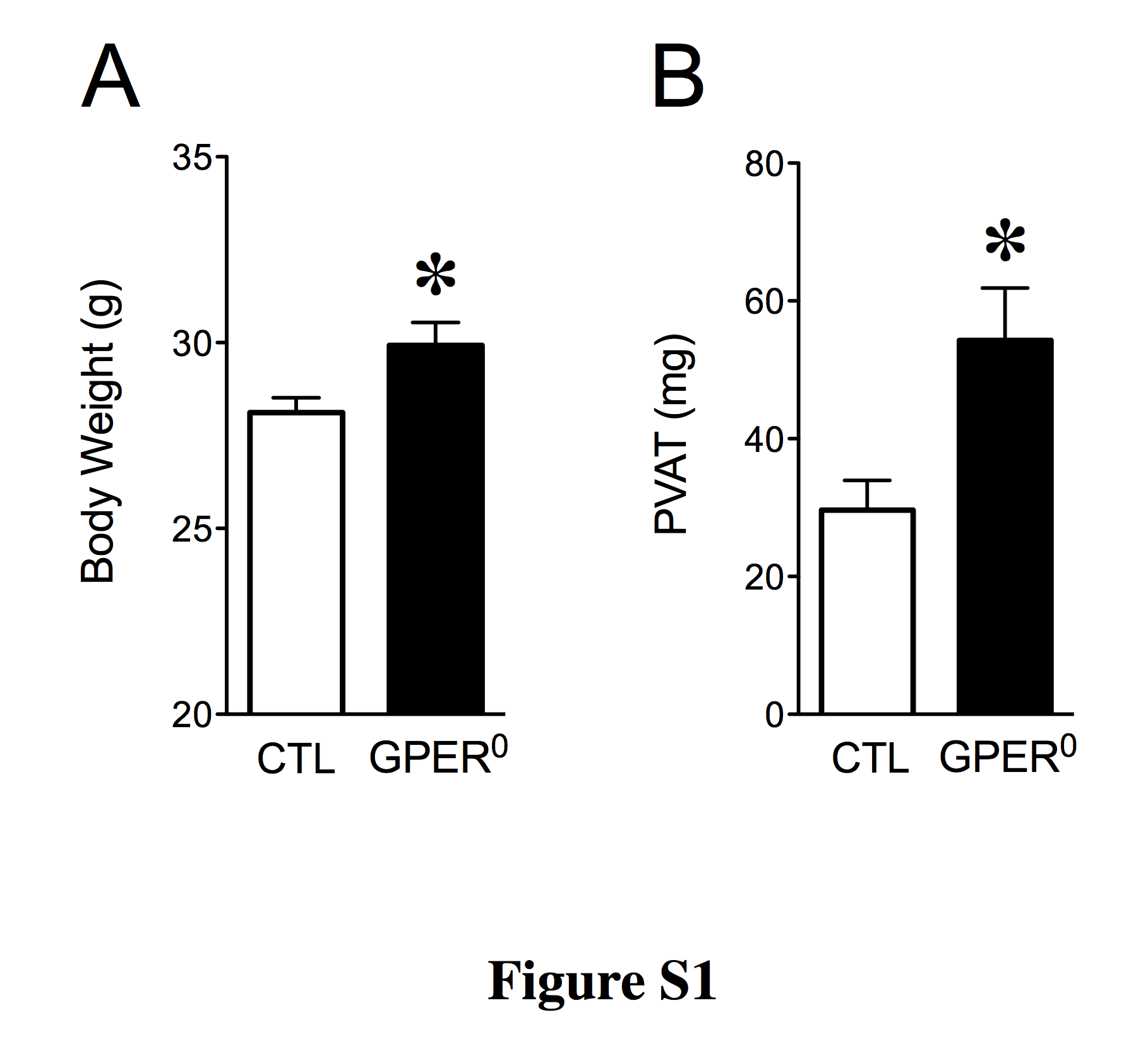

Supplement: Figure S1 — Obesity in 3 month-old male GPER0 mice. A, Body weight of 3 month-old male GPER0 mice (, n = 16) and WT controls (CTL, , n = 27). *p = 0.01 vs. CTL. B, Aortic perivascular adipose (PVAT) mass of 3 month-old male GPER0 (, n = 5) and CTL mice (CTL, , n = 5). *p = 0.02 vs. CTL. (TIFF) [file pone.0079245.s001.tiff]

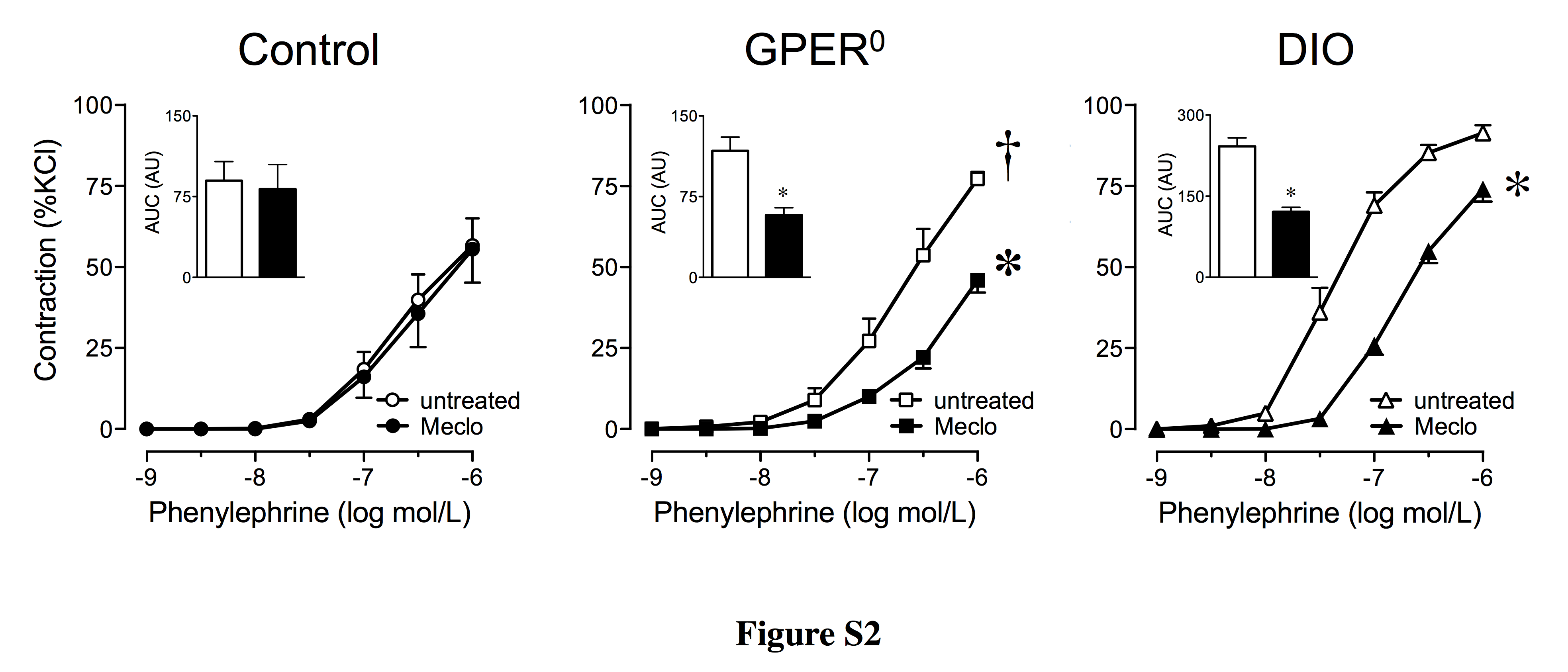

Supplement: Figure S2 — COX inhibition of phenylephrine-induced contractions in vessels of obese mice. Aortic rings with perivascular adipose were obtained from WT mice fed a regular chow (control), GPER0 mice, and WT animals fed a high-fat diet (DIO). Where indicated, rings were treated with the cyclooxygenase inhibitor meclofenamate (Meclo, 1 mmol/L) prior to stimulation. Inset: Area under the curve (AUC) is expressed as arbitrary units (AU). , control, untreated (n = 6); , control, meclofenamate (n = 7); , GPER0, untreated (n = 6); GPER0, meclofenamate, (n = 6); , DIO, untreated (n = 6); , DIO, meclofenamate (n = 6). *p<0.01 vs. untreated vascular rings; †P<0.05 vs. control. (TIFF) [file pone.0079245.s002.tiff]

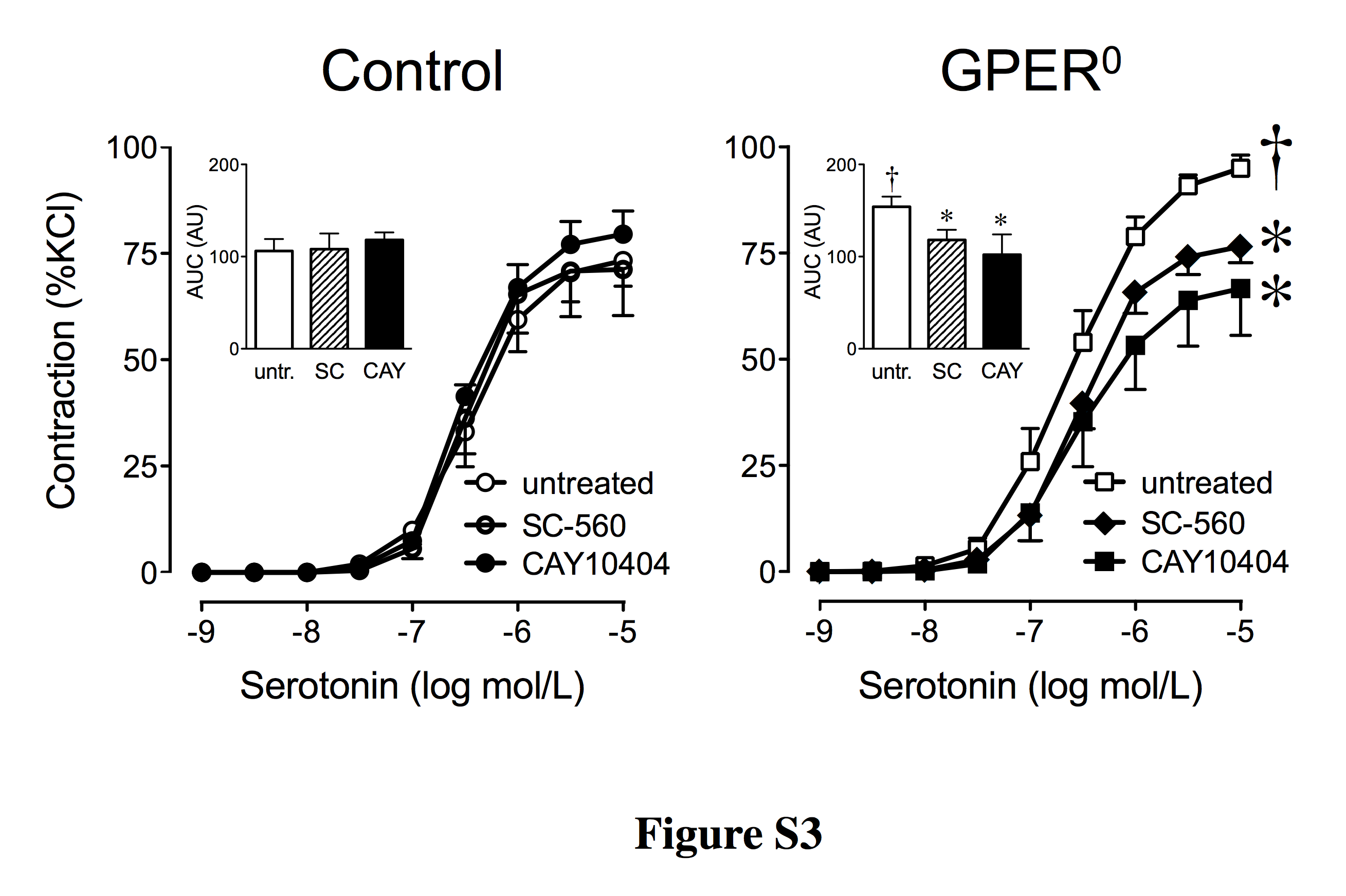

Supplement: Figure S3 — Cyclooxygenase subtype-specific inhibition of vascular contraction in mice with monogenic obesity. Aortic rings with perivascular adipose were obtained from GPER0 mice and WT mice fed a regular chow (control). Concentration-dependent contractions to serotonin were determined in the presence of selective inhibitors for cyclooxygenase type 1 (SC-560, 300 nmol/L) and type 2 (CAY10404, 100 nmol/L). Inset: Area under the curve (AUC) is expressed as arbitrary units (AU). , control, untreated (n = 6); , control, SC-560 (n = 4); , control, CAY10404 (n = 4); , GPER0, untreated (n = 7); , GPER0, SC-560 (n = 7); GPER0, CAY10404 (n = 6). *p<0.05 vs. untreated vascular rings; †p<0.05 vs. control. (TIFF) [file pone.0079245.s003.tiff]

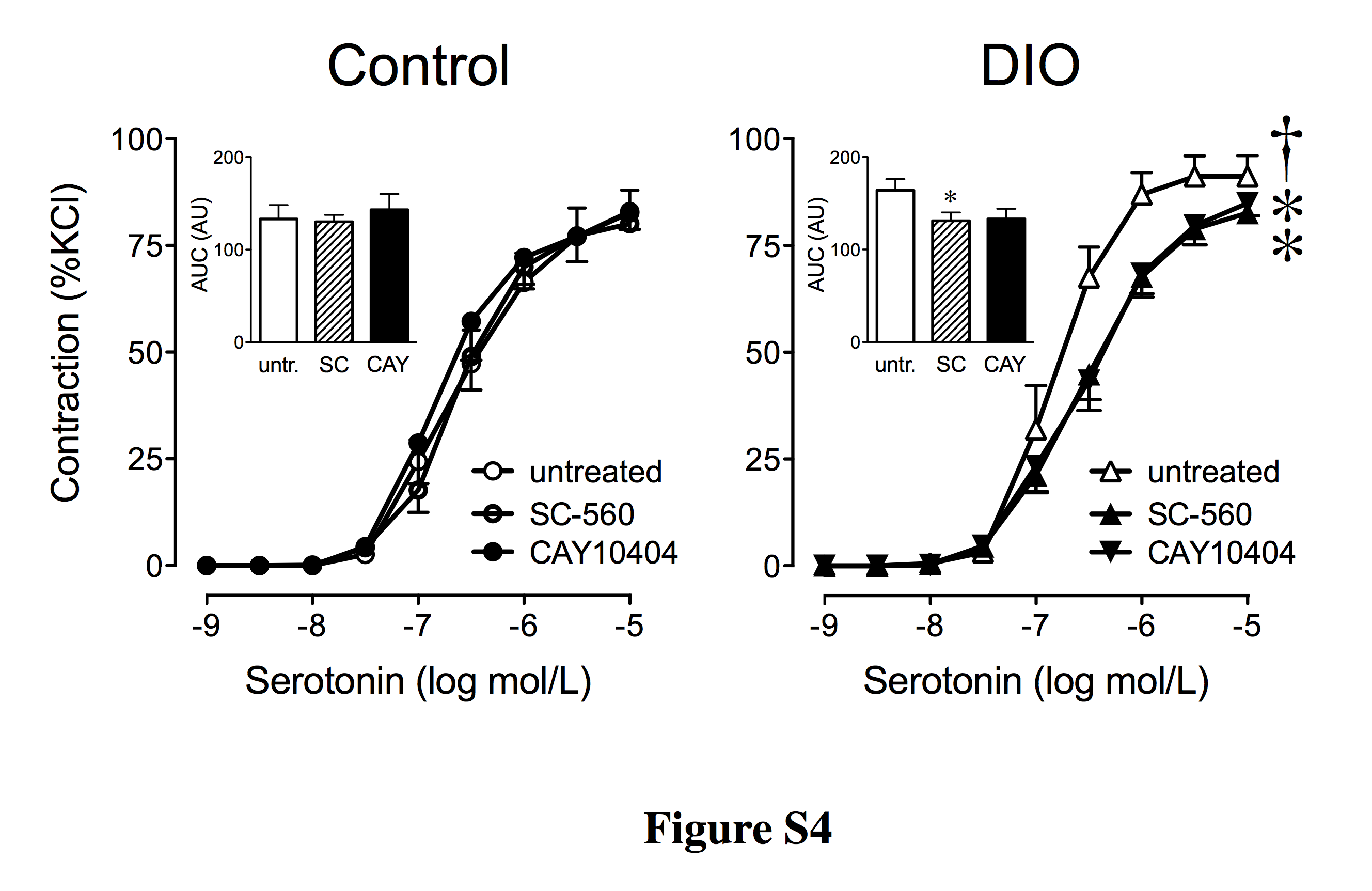

Supplement: Figure S4 — Cyclooxygenase subtype-specific inhibition of vascular contraction in perivascular adipose-intact aortic rings of mice with diet-induced obesity. Animals were fed a high-fat diet for 24 weeks (DIO) and compared to age-matched mice fed standard chow (control). Concentration-dependent contractions to serotonin were determined in the presence of selective inhibitors for cyclooxygenase type 1 (SC-560, 300 nmol/L) and type 2 (CAY10404, 100 nmol/L). Inset: Area under the curve (AUC) is expressed as arbitrary units (AU). , control, untreated (n = 6); , control, SC-560 (n = 4); , control, CAY10404 (n = 4); , DIO, untreated (n = 7); , DIO, SC-560 (n = 6); , DIO, CAY10404 (n = 5). *p<0.05 vs. untreated vascular rings; †p<0.05 vs. control. (TIFF) [file pone.0079245.s004.tiff]
