# Supplementary material for: Regulation of Vascular Smooth Muscle Tone by Adipose-Derived Contracting Factor
Source: PLoS One. 2013 Nov 11;8(11):e79245. doi: 10.1371/journal.pone.0079245 (PMC3823600; doi:10.1371/journal.pone.0079245)
Supplement: Table S1 — Sets of primers used for amplification of gene-specific cDNA fragments by qPCR. TNFα, tumor necrosis factor α; PPARγ, peroxisome proliferator activated receptor γ; COX, cyclooxygenase. (DOC) [file pone.0079245.s005.doc]

Supplemental Table

| Gene  (Accession Number) | Forward Primer | Reverse Primer |
| --- | --- | --- |
| Adiponectin (NM_009605) | 5’-GAT GGC AGA GAT GGC ACT CC-3′ | 5’-CTT GCC AGT GCT GCC GTC AT-3′ |
| Leptin (NM_008493) | 5’-AGA CCG GGA AAG AGT G-3’ | 5’- GCC ATA GTG CAA GGT T-3’ |
| TNFα (NM013693) | 5’-TCC CAG GTT CTC TTC AAG GGA-3′ | 5’-GGT GAG GAG CAC GTA GTC GG-3′ |
| PPARγ (NM001127330) | 5'-GAT GGA AGA CCA CTC GCA TT-3' | 5'-AAC CAT TGG GTC AGC TCT TG-3' |
| COX-1 (NM_008969) | 5’-ACT CAG CGC ATG ACT ACA TC-3’ | 5’-CTT CTC AGC AGC AGC TGT TG-3’ |
| COX-2 (NM_011198) | 5’-GAA CAT GGA CTC ACT CAG TTT GTT G-3’ | 5’-CAA AGA TAG CAT CTG GAC GAG GT-3’ |
| GAPDH (NM_008084) | 5’-GAG CCA AAC GGG TCA TCA-3’ | 5’-CAT ATT TCT CGT GGT TCA CAC-3’ |

**Supplemental Table S1.** Sets of primers used for amplification of gene-specific cDNA fragments by qPCR. TNFα, tumor necrosis factor α; PPARγ, peroxisome proliferator activated receptor gamma; COX, cyclooxygenase.
